# Supplementary material for: biomonitoR: an R package for managing ecological data and calculating biomonitoring indices
Source: PeerJ. 2022 Oct 14;10:e14183. doi: 10.7717/peerj.14183 (PMC11566503; doi:10.7717/peerj.14183)
Supplement: Supplemental Information 2 [file peerj-10-14183-s002.docx]

**Supplementary Materials 1-2**

**For any question or bug reporting please drop a mail to** [**alex.laini@unito.it**](mailto:alex.laini@unito.it)**,** [**s.guareschi@ebd.csic.es**](mailto:s.guareschi@ebd.csic.es)

**- Article**

**biomonitoR: an R package for managing ecological data and calculating biomonitoring indices**

**- Authors**

Alex Laini^1,2^, Simone Guareschi^3,4^, Rossano Bolpagni^1^, Gemma Burgazzi^5^, Daniel Bruno^6^, Cayetano Gutiérrez-Cánovas^4^, Rafael Miranda^7^, Cédric Mondy^8^, Gábor Várbíró^9^, Tommaso Cancellario^7,10^

^1^Department of Chemistry, Life Sciences and Environmental Sustainability, University of Parma, Italy

^2^Department of Life Sciences and Systems Biology, University of Turin, Italy

^3^Geography and Environment, Loughborough University, Leicestershire, UK

^4^Estación Biológica de Doñana (EBD), CSIC, Seville, Spain.

^5^Quantitative Landscape Ecology, Institute for Environmental Sciences, University of Koblenz-Landau, Landau, Germany

^6^Departamento de Conservación de la Biodiversidad y Restauración de Ecosistemas, Instituto Pirenaico de Ecología (IPE), CSIC, Zaragoza, Spain

^7^Department of Environmental Biology, University of Navarra, Pamplona, Spain

^8^French Agency for Biodiversity (OFB), Vincennes, France

^9^Centre for Ecological Research, Institute of Aquatic Ecology, Debrecen, Hungary

^10^Water Research Institute, National Research Council (CNR), Verbania, Italy

**SM1 – Reference datasets in biomonitoR**

The reference taxonomic dataset is fundamental to get the most from biomonitoR. It allows to i) calculate most indices at different taxonomic level effortless and ii) to check for misspelled taxa names and to suggest correct names. biomonitoR comes with three ways to construct a reference dataset.

**Built-in taxonomic datasets**

Four datasets for diatoms, macrophytes, macroinvertebrates and fish are currently available.

- **Diatoms:** Diat.barcode-release-version 10.1.xlsx (2021-06-25) (Rimet et al., 2019).
- **Macrophytes:** taxonomic information was retrieved from freshwaterecology.info version 7.0 and modified to fit with the biomonitoR format. Species aggregation, undescribed species, lineages were removed to better fit with biomonitoR. Synonyms were replaced with accepted names.
- **Macroinvertebrates:** taxonomic information was retrieved from freshwaterecology.info version 7.0 and modified to fit with the biomonitoR format. Species aggregation, undescribed species, lineages were removed to better fit with biomonitoR.
- **Fish:** Fishbase version 18.07 with additional checks using the Eschmeyer dataset (https://www.calacademy.org/scientists/projects/catalog-of-fishes).

**Functions to retrieve taxonomic information from online resources**

There are currently four functions to retrieve taxonomic information from online resources for any biotic group. New functions will be added in the future targeting online resources different from those already implemented.

- get_gbif_taxa_tree: it relies on the Global Biodiversity Information Facility (GBIF, <https://www.gbif.org/>). This function automatically replaces synonyms with accepted names.
- get_iucn_taxa_tree: it relies on the International Union for Conservation of Nature (IUCN) red list of threatened species (https://www.iucnredlist.org/). Its usage is recommended for taxa included in the Red Lists. This function necessitates of token to work properly.
- get_nbn_taxa_tree: it relies on the National Biodiversity Network of UK (NBN, <https://nbnatlas.org/>).
- get_worms_taxa_tree: it relies on the World Register of Marine Species (WoRMS, <https://www.marinespecies.org>).

**Users’ datasets**

**Taxonomic reference dataset**

Users can build their own reference dataset in the biomonitoR format using a standard taxonomic tree and the function ref_from_tree. See supplementary material 3 and the function documentation for a usage example.

**Functional information dataset**

See Supplementary Material 3 (R File) for usage examples from Poff et al. 2006 (trait information from North American insects) and Sarremejane et al. 2020 (European aquatic macroinvertebrates).

**References**

Fricke, R., Eschmeyer, W. N. & R. van der Laan (eds) 2022. Eschmeyer's catalog of fishes: genera, species, references. Electronic version accessed 09-11-2018. <http://researcharchive.calacademy.org/research/ichthyology/catalog/fishcatmain.asp>

Poff, N.L., Olden, J.D., Vieira, N.K., Finn, D.S., Simmons, M.P. and Kondratieff, B.C., 2006. Functional trait niches of North American lotic insects: traits-based ecological applications in light of phylogenetic relationships. Journal of the North American Benthological Society, 25(4), pp.730-755. [https://www.journals.uchicago.edu/doi/10.1899/0887-3593(2006)025[0730:FTNONA]2.0.CO;2](https://www.journals.uchicago.edu/doi/10.1899/0887-3593(2006)025%5b0730:FTNONA%5d2.0.CO;2)

Rimet F., Gusev E., Kahlert M., Kelly M., Kulikovskiy M., Maltsev Y., Mann D., Pfannkuchen M., Trobajo R., Vasselon V., Zimmermann J., Bouchez A., 2019. Diat.barcode, an open-access curated barcode library for diatoms. Scientific Reports, 9: 15116. <https://www.nature.com/articles/s41598-019-51500-6>

Sarremejane, R., Cid, N., Stubbington, R., Datry, T., Alp, M., Cañedo-Argüelles, M., Cordero-Rivera, A., Csabai, Z., Gutiérrez-Cánovas, C., Heino, J. and Forcellini, M., Millán, A., Paillex, A., Pařil, P., Polášek, M., Tierno de Figueroa, J.M., Usseglio-Polatera, P., Zamora-Muñoz C. & Bonada, N. 2020. DISPERSE, a trait database to assess the dispersal potential of European aquatic macroinvertebrates. Scientific Data, 7: 386. <https://www.nature.com/articles/s41597-020-00732-7>

**Table SM2.** List of the main metrics available in biomonitoR. Target group, aims, methodologic refences and examples of applications are also provided. SEA= Supporting Ecological Assessment; BD=Biodiversity assessment; SP=Single Pressure: MI= Macroinvertebrates; MP= Macrophytes.

Metric glossary: BMWP=Biological Monitoring Working Party; ASPT=Average Score Per Taxon; IBMWP=Iberian Biological Monitoring Working Party; IASPT= Iberian Average Score Per Taxon; EPT=Ephemeroptera, Plecoptera, Trichoptera; Log_10_(SEL_EPTD+1) = Logarithm of the selected families of Ephemeroptera, Plecoptera, Trichoptera and Diptera plus 1; 1-GOLD= 1 minus the relative abundance of Gastropoda, Oligochaeta and Diptera; WHPT= Walley, Hawkes, Paisley and Trigg; IBMR= Macrophyte Biological Index for Rivers; LIFE= Lotic-invertebrate Index for Flow Evaluation; PSI= Proportion of Sediment-sensitive Invertebrates; EPSI= Empirically-weighted PSI; ACI= Abundance Contamination Index; RCI= Richness Contamination Index; SBCI= Site-specific Biocontamination Index.

| **Index/metric** | **Target group** | **Aims** | biomonitoR  **function** | **Methodologic reference** | **Index application examples** |
| --- | --- | --- | --- | --- | --- |
| TOTAL TAXA | All components | SEA /BD | richness,  allrich | Buss et al. 2015 | Italy, Morocco, Spain: Gutiérrez-Cánovas et al. 2019 |
| BMWP | MI | SEA | bmwp | Hawkes 1998 | UK: Clarke et al. 2002 |
| ASPT | MI | SEA | aspt | Hawkes 1998 | UK: Clarke et al. 2002 |
| IBMWP | MI | SEA | bmwp | Alba Tercedor et al. 2002 | Spain: Bruno et al. 2014 |
| IASPT | MI | SEA | aspt | Alba Tercedor et al. 2002 | Spain and Italy: Guareschi et al. 2017 |
| EPT | MI | SEA | ept, get_taxa_richness | Kitchin 2005 | Australia: Wright and Ryan, 2016 |
| Log_10_(SEL_EPTD+1) | MI | SEA | eptd | Erba et al. 2006 | Spain: Munné and Prat, 2009  Italy: Laini et al. 2018. |
| 1-GOLD | MI | SEA | igold | Pinto et al. 2004 | Italy: Laini et al. 2018. |
| WHPT | MI | SEA | whpt | Paisley et al. 2014 | UK: Guareschi et al. 2021a |
| WHPT-ASPT | MI | SEA | whpt | Paisley et al. 2014 | UK: Guareschi et al. 2021a |
| IBMR | MP | SEA | ibmr | Haury et al. 2006 | Cyprus, France, Greece, Italy, Portugal, Spain: Aguiar et al. 2014 |
| LIFE | MI | SP: flow variation | life | Extence et al. 1999 | UK: Monk et al. 2008 |
| Flow-T | MI | SP: flow variation | fuzzy_trait_ratio | Laini et al. 2022 | UK, Italy, Cyprus: Laini et al. 2022 |
| DEHLI | MI | SP: drought effects | dehli | Chadd et al. 2017 | UK: Chadd et al. 2017 |
| PSI | MI | SP: sedimentation | psi | Extence et al. 2013 | UK: Extence et al. 2017 |
| EPSI | MI | SP: sedimentation | epsi | Turley et al. 2016 | UK: Wilkes et al. 2017 |
| RCI | All components | SP: biocontamination | bioco | Arbačiauskas et al. 2008 | UK: Guareschi et al. 2021b |
| ACI | All components | SP: biocontamination | bioco, get_taxa_abundance | Arbačiauskas et al. 2008 | UK: Guareschi et al. 2021b |
| SBCI | All components | SP: biocontamination | bioco | Arbačiauskas et al. 2008 | Russia: Son et al. 2020 |
| Functional Richness | All components | BD: functional | f_rich | Villeger et al. 2008 | Spain: Belmar et al. 2019 |
| Functional Redundancy | All components | BD: functional | f_red | Pillar et al. 2013 | Spain: Bruno et al. 2016 |
| Functional Diversity | All components | BD: functional | f_divs | Petchey and Gaston 2006 | UK: Mathers et al. 2020 |
| Functional Dispersion | All components | BD: functional | f_disp | Villeger et al. 2008 | UK: Mathers et al. 2020 |
| Functional Evenness | All components | BD: functional | f_eve | Villeger et al. 2008 | UK: Mathers et al. 2020 |
| Community trait specialization | All components | BD: functional | csi | Mondy and Usseglio-Polatera 2013 | France, Spain:  Dolédec et al. 2017 |
| Shannon | All components | BD: taxonomic | shannon, allindices | Shannon 1949 | Spain: Mellado et al. 2019 |
| Pielou index | All components | BD: taxonomic | pielou, allindices | Pielou 1966 | France: Biesel et al. 2003  Finland: Heino et al. 2007 |
| Margalef Diversity | All components | BD: taxonomic | margalef, allindices | Magurran and McGill 2010 | France:  Thiebaut et al. 2006  Ireland:  Gray and Delaney 2008 |
| Menhinick Diversity | All components | BD: taxonomic | menhinick, allindices | Magurran and McGill 2010 | Ireland:  Gray and Delaney 2008 |
| Brillouin index | All components | BD: taxonomic | brillouin, allindices | Magurran 2004 | Ireland:  Gray and Delaney 2008 |
| Simpson index | All components | BD: taxonomic | simpson, invsimpson, allindices | Magurran 2004 | France:  Thiebaut et al. 2006  Ireland:  Gray and Delaney 2008 |
| Simpson evenness | All components | BD: taxonomic | esimpson, allindices | Magurran 2004 | Netherlands: Verdonschot et al. 2012 |
| Berger-Parker index | All components | BD: taxonomic | berpar, invberpar, allindices | Magurran 2004 | UK: Wood et al. 2005 |
| McIntosh diversity | All components | BD: taxonomic | mcintosh, allindices | Magurran 2004 | France: Guerold 2000 |
| Fisher alpha | All components | BD: taxonomic | fisher, allindices | Magurran 2004 | Switzerland: Burdon et al., 2016 |
| Taxonomic diversity | All components | BD: taxonomic/phylogenetic | dness | Clarke and Warwick 1998 | Finland: Heino et al. 2007 |
| Taxonomic distinctness | All components | BD: taxonomic/phylogenetic | dness | Clarke and Warwick 1998 | Finland: Heino et al. 2005, Heino et al. 2007 |
| Variation of taxonomic distinctness | All components | BD: taxonomic/phylogenetic | dness | Clarke and Warwick 2001 | Ireland: Leira et al. 2009  Finland: Heino et al. 2007 |

**References used here:**

Aguiar, F.C., Segurado, P., Urbanič, G., Cambra, J., Chauvin, C., Ciadamidaro, S., Dörflinger, G., Ferreira, J., Germ, M., Manolaki, P., Minciardi, M.R., Munné, A., Papastergiadou, E., Ferreira, M.T., 2014. Comparability of river quality assessment using macrophytes: A multi-step procedure to overcome biogeographical differences. Science of The Total Environment, 476, 757-767.

Alba Tercedor, J., Jáimez-Cuéllar, P., Álvarez, M., Avilés, J., Bonada i Caparrós, N., Casas, J., Mellado, A., Ortega, M., Pardo, I., Prat i Fornells, N., Rieradevall i Sant, M., 2002. Caracterización del estado ecológico de ríos mediterráneos ibéricos mediante el índice IBMWP (antes BMWP'). Limnetica, 2002, 21 (3-4), 175-185.

Arbačiauskas, K., Semenchenko, V., Grabowski, M., Leuven, R.S.E.W., Paunović, M., Son, M., Csanyi, B., Gumuliauskaitė, S., Konopacka, A., Nehring, S., van der Velde, G., Vezhnovetz, V., Panov, V.E. 2008. Assessment of biocontamination of benthic macroinvertebrate communities in European inland waterways. Aquatic Invasions 3, 211–230.

Beisel, J.N., Usseglio‐Polatera, P., Bachmann, V., Moreteau, J.C., 2003. A comparative analysis of evenness index sensitivity. International Review of Hydrobiology: A Journal Covering all Aspects of Limnology and Marine Biology, 88(1), 3-15.

Belmar, O., Bruno, D., Guareschi, S., Mellado‐Díaz, A., Millán, A., Velasco, J., 2019. Functional responses of aquatic macroinvertebrates to flow regulation are shaped by natural flow intermittence in Mediterranean streams. Freshwater Biology, 64(5), 1064-1077.

Bruno, D., Belmar, O., Sánchez-Fernández, D., Guareschi, S., Millán, A., Velasco, J. 2014. Responses of Mediterranean aquatic and riparian communities to human pressures at different spatial scales. Ecological Indicators, 45, 456-464.

Bruno, D., Gutiérrez-Cánovas, C., Velasco, J., Sánchez-Fernández, D., 2016. Functional redundancy as a tool for bioassessment: A test using riparian vegetation. Science of the Total Environment, 566, 1268-1276.

Burdon, F.J., Reyes, M., Alder, A.C., Joss, A., Ort, C., Räsänen, K., Jokela, J., Eggen, R.I.L., Stamm, C. 2016. Environmental context and magnitude of disturbance influence trait‐mediated community responses to wastewater in streams. Ecology and Evolution, 6(12), 3923-3939.

Buss, D.F., Carlisle, D.M., Chon, T.S., Culp, J., Harding, J.S., Keizer-Vlek, H.E., Robinson, W.A., Strachan, S., Thirion, C., Hughes, R.M., 2015. Stream biomonitoring using macroinvertebrates around the globe: a comparison of large-scale programs. Environmental Monitoring and Assessment, 187(1), 1-21.

Chadd, R.P., England, J.A., Constable, D., Dunbar, M.J., Extence, C.A., Leeming, D.J., Murray-Bligh, J.A., Wood, P.J., 2017. An index to track the ecological effects of drought development and recovery on riverine invertebrate communities. Ecological Indicators, 82, pp.344-356.

Clarke, R.T., Furse, M.T., Gunn, R.J.M., Winder, J.M., Wright, J.F., 2002. Sampling variation in macroinvertebrate data and implications for river quality indices. Freshwater Biology, 47(9), 1735-1751.

Clarke, K.R., Warwick, R.M. 1998. A taxonomic distinctness index and its statistical properties. Journal of Applied Ecology, 35(4), 523-531.

Clarke, K.R., Warwick, R.M. 2001. A further biodiversity index applicable to species lists: variation in taxonomic distinctness. Marine ecology Progress series, 216, 265-278.

Dolédec, S., Tilbian, J., Bonada, N. 2017. Temporal variability in taxonomic and trait compositions of invertebrate assemblages in two climatic regions with contrasting flow regimes. Science of the Total Environment, 599, 1912-1921.

Erba, S., Buffagni, A., Holmes, N., O’Hare, M., Scarlett, P., Stenico, A. 2006. Preliminary testing of River Habitat Survey features for the aims of the WFD hydro-morphological assessment: an overview from the STAR Project. Hydrobiologia, 566(1), 281-296.

Extence, C.A., Balbi, D.M., Chadd, R.P., 1999. River flow indexing using British benthic macroinvertebrates: a framework for setting hydroecological objectives. Regulated Rivers: Research & Management, 15(6), 545-574.

Extence, C.A, Chadd, R., England, J., Dunbar, M.J., Wood, P.J., D. Taylor, E., 2013. The assessment of fine sediment accumulation in rivers using macro‐invertebrate community response. River Research and Applications, 29(1), 17-55.

Extence, C.A., Chadd, R.P., England, J., Naura, M., Pickwell, A.G.G., 2017. Application of the proportion of sediment‐sensitive invertebrates (PSI) biomonitoring index. River Research and Applications, 33(10), 1596-1605.

Gray, N.F., Delaney, E. 2008. Comparison of benthic macroinvertebrate indices for the assessment of the impact of acid mine drainage on an Irish river below an abandoned Cu–S mine. Environmental Pollution, 155(1), 31-40.

Guareschi, S., Laini, A., Sanchez-Montoya, M.M., 2017. How do low-abundance taxa affect river biomonitoring? Exploring the response of different macroinvertebrate-based indices. Journal of Limnology, 76, 9-20.

Guareschi, S, Laini, A, England, J, Johns, T, Winter, M, Wood PJ. 2021a. Invasive species influence macroinvertebrate biomonitoring tools and functional diversity in British rivers. Journal of Applied Ecology, 58,135-147.

Guareschi, S., Laini, A., England, J., Barrett, J., Wood, P.J. 2021b. Multiple co‐occurrent alien invaders constrain aquatic biodiversity in rivers. Ecological Applications, 31(6), e02385.

Guerold, F. 2000. Influence of taxonomic determination level on several community indices. Water Research, 34(2), 487-492.

Gutiérrez-Cánovas, C., Arribas, P., Naselli-Flores, L., Bennas, N., Finocchiaro, M., Millán, A., Velasco, J., 2019. Evaluating anthropogenic impacts on naturally stressed ecosystems: Revisiting river classifications and biomonitoring metrics along salinity gradients. Science of the Total Environment, 658, 912-921.

Haury, J., Peltre, M.C., Trémolières, M., Barbe, J., Thiébaut, G., Bernez, I., Daniel, H., Chatenet, P., Haan-Archipof, G.,Muller, S., Dutartre, A., Laplace-Treyture, C., Cazaubon, A., Lambert-Servien, E., 2006. A new method to assess water trophy and organic pollution – the Macrophyte Biological Index for Rivers (IBMR): its application to different types of river and pollution. Hydrobiologia 570, 153–158.

Hawkes, H.A., 1998. Origin and development of the biological monitoring working party score system. Water Research, 32(3), 964-968.

Heino, J., Soininen, J., Lappalainen, J., Virtanen, R., 2005. The relationship between species richness and taxonomic distinctness in freshwater organisms. Limnology and Oceanography, 50(3), 978-986.

Heino, J., Mykrä, H., Hämäläinen, H., Aroviita, J., Muotka, T. 2007. Responses of taxonomic distinctness and species diversity indices to anthropogenic impacts and natural environmental gradients in stream macroinvertebrates. Freshwater Biology, 52(9), 1846-1861.

Kitchin, P.L., 2005. Measuring the amount of statistical information in the EPT index. Environmetrics: The official journal of the International Environmetrics Society, 16(1), 51-59.

Laini, A., Bolpagni, R., Cancellario, T., Guareschi, S., Racchetti, E., Viaroli, P., 2018. Testing the response of macroinvertebrate communities and biomonitoring indices under multiple stressors in a lowland regulated river. Ecological Indicators, 90, 47-53.

Laini, A., Burgazzi, G., Chadd, R., England, J., Tziortzis, I., Ventrucci, M., Vezza, P., Wood, P.J., Viaroli, P., Guareschi, S., 2022. Using invertebrate functional traits to improve flow variability assessment within European rivers. Science of The Total Environment, 832, 155047.

Leira, M., Chen, G., Dalton, C., Irvine, K., Taylor, D., 2009. Patterns in freshwater diatom taxonomic distinctness along an eutrophication gradient. Freshwater Biology, 54(1), 1-14.

Magurran, A. E., 2004. Measuring biological diversity. Blackwell Science ltd.

Magurran, A.E., McGill, B.J. 2010. Biological diversity: frontiers in measurement and assessment. OUP Oxford.

Mathers, K.L., White, J.C., Guareschi, S., Hill, M.J., Heino, J., Chadd, R., 2020. Invasive crayfish alter the long‐term functional biodiversity of lotic macroinvertebrate communities. Functional Ecology, 34(11), 2350-2361.

Mellado-Díaz, A., Sánchez-González, J.R., Guareschi, S., Magdaleno, F., Velasco, M.T., 2019. Exploring longitudinal trends and recovery gradients in macroinvertebrate communities and biomonitoring tools along regulated rivers. Science of the Total Environment, 695, p.133774.

Mondy, C.P., Usseglio-Polatera P., 2013. Using Fuzzy-Coded Traits to Elucidate the Non-Random Role of Anthropogenic Stress in the Functional Homogenisation of Invertebrate Assemblages. Freshwater Biology, 59 (3), 584-600.

Monk, W.A., Wood, P.J., Hannah, D.M., Wilson, D.A., 2008. Macroinvertebrate community response to inter‐annual and regional river flow regime dynamics. River Research and Applications, 24(7), 988-1001.

Munné, A., Prat, N., 2009. Use of macroinvertebrate-based multimetric indices for water quality evaluation in Spanish Mediterranean rivers: an intercalibration approach with the IBMWP index. Hydrobiologia, 628(1), 203-225

Nichols, S., Weber, S., Shaw, B., 2000. A proposed aquatic plant community biotic index for Wisconsin lakes. Environmental Management, 26(5), 491-502.

Paisley, M.F., Trigg, D.J., Walley, W.J., 2014. Revision of the Biological Monitoring Working Party (BMWP) score system: derivation of present‐only and abundance‐related scores from field data. River Research and Applications, 30(7), 887-904.

Petchey, O.L., Gaston, K.J., 2006. Functional diversity: back to basics and looking forward. Ecology Letters, 9(6), 741-758.

Pielou E.C., 1966. The measurement of diversity in different types of biological collections. Journal of Theoretical Biology., 13, 131–144.

Pinto, P., Rosado, J., Morais, M., Antunes, I., 2004. Assessment methodology for southern siliceous basins in Portugal. In Integrated Assessment of Running Waters in Europe (pp. 191-214). Springer, Dordrecht.

Shannon, C.E., 1948. A mathematical theory of communication. Bell System Technical Journal, 27, 379–423.

Simpson, W. 1949. Measurement of diversity. Nature 163:688.

Son, M.O., Prokin, A.A., Dubov, P.G., Konopacka, A., Grabowski, M., MacNeil, C., Panov, V.E., 2020. Caspian invaders vs. Ponto-Caspian locals–range expansion of invasive macroinvertebrates from the Volga Basin results in high biological pollution of the Lower Don River. Management of Biological Invasions, 11(2), 178.

Thiebaut, G., Tixier, G., Guerold, F., Muller, S. 2006. Comparison of different biological indices for the assessment of river quality: application to the upper river Moselle (France). Hydrobiologia, 570(1), 159-164.

Turley, M.D., Bilotta, G.S., Chadd, R.P., Extence, C.A., Brazier, R.E., Burnside, N.G., Pickwell, A.G., 2016. A sediment-specific family level biomonitoring tool to identify the impacts of fine sediment in temperate rivers and streams. Ecological Indicators, 70, 151–165.

Verdonschot, R.C., Keizer-Vlek, H.E., Verdonschot, P.F., 2012. Development of a multimetric index based on macroinvertebrates for drainage ditch networks in agricultural areas. Ecological Indicators, 13(1), 232-242.

Wilkes, M.A., Mckenzie, M., Murphy, J.F., Chadd, R.P., 2017. Assessing the mechanistic basis for fine sediment biomonitoring: Inconsistencies among the literature, traits and indices. River Research and Applications, 33(10), 1618-1629.

Wood, P.J., Gunn, J., Smith, H., Abas-Kutty, A., 2005. Flow permanence and macroinvertebrate community diversity within groundwater dominated headwater streams and springs. Hydrobiologia, 545(1), 55-64.

Wright, I.A. and Ryan, M.M., 2016. Impact of mining and industrial pollution on stream macroinvertebrates: importance of taxonomic resolution, water geochemistry and EPT indices for impact detection. Hydrobiologia, 772(1), 103-115.
