# Supplementary material for: biomonitoR: an R package for managing ecological data and calculating biomonitoring indices
Source: PeerJ. 2022 Oct 14;10:e14183. doi: 10.7717/peerj.14183 (PMC11566503; doi:10.7717/peerj.14183)
Supplement: Supplemental Information 3 [file peerj-10-14183-s003.docx]

**Supplementary Material 4**

**- Article**

**biomonitoR: an R package for managing ecological data and calculating biomonitoring indices**

**- Authors:** Alex Laini, Simone Guareschi, Rossano Bolpagni, Gemma Burgazzi, Daniel Bruno, Cayetano Gutiérrez-Cánovas, Rafael Miranda, Cédric Mondy, Gábor Várbíró, Tommaso Cancellario

**SM4 – Default plots in biomonitoR**

The biomonitoR package relies on the plotly (Sievert, 2020) package for generating high quality and interactive plots. The following plots were generated from a dataset of aquatic insects identified at mixed taxonomic level and collected from 8 sites in Northern Italy in June 2019 (40 observations). Examples reported here (S4a, S4b, S4c, S4d) can be reproduced with the R code available below.


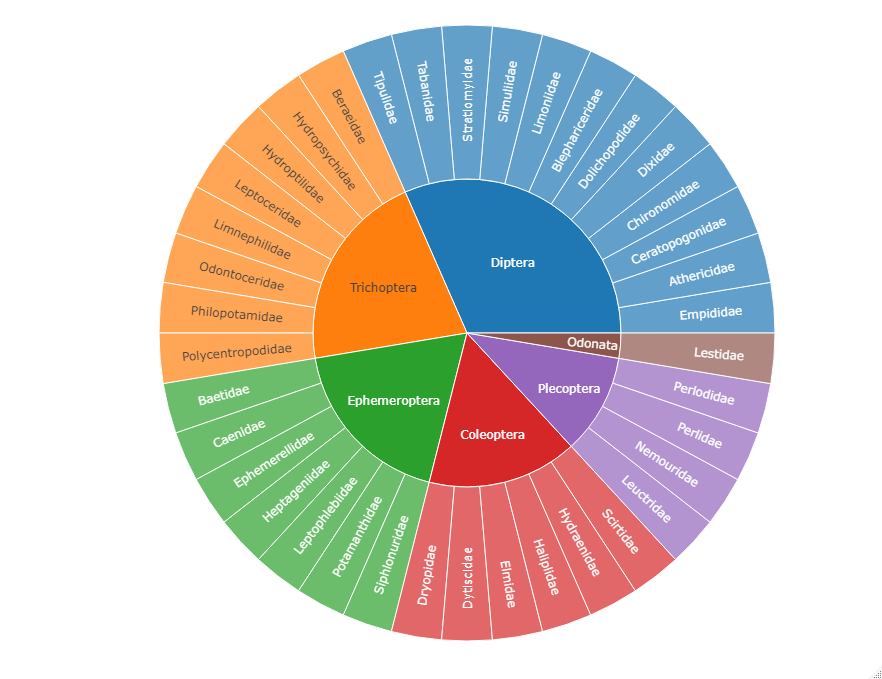


**Figure S4a** Overall structure of an aquatic insect community. Presence-absence data are reported at Family level.


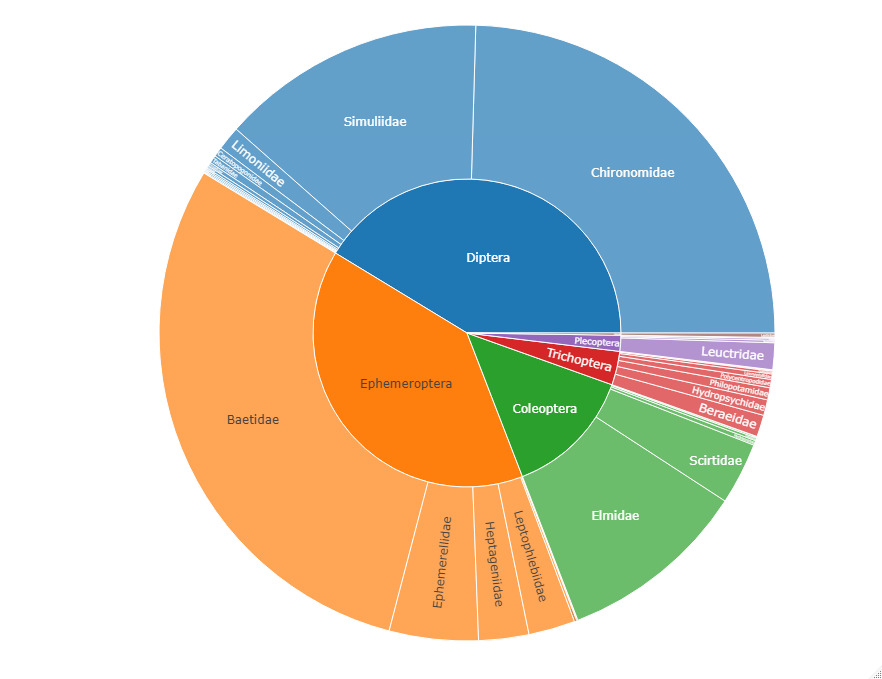


**Figure S4b** Overall structure of an aquatic insect community. Abundance data are reported at family level.


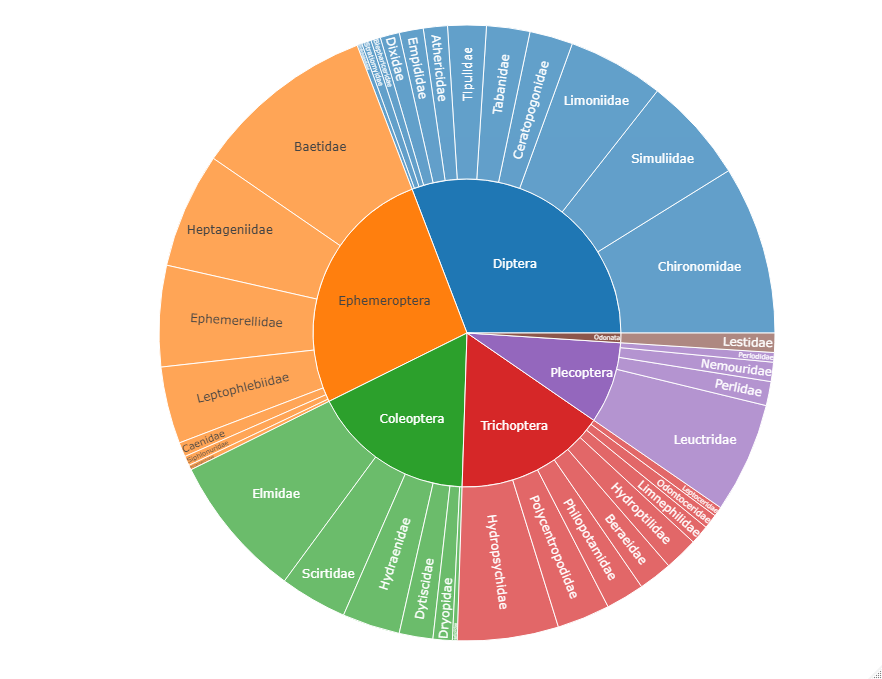


**Figure S4c** Overall structure of an aquatic insect community. Frequency data (the number of sample where a taxon was detected) are reported at family level.


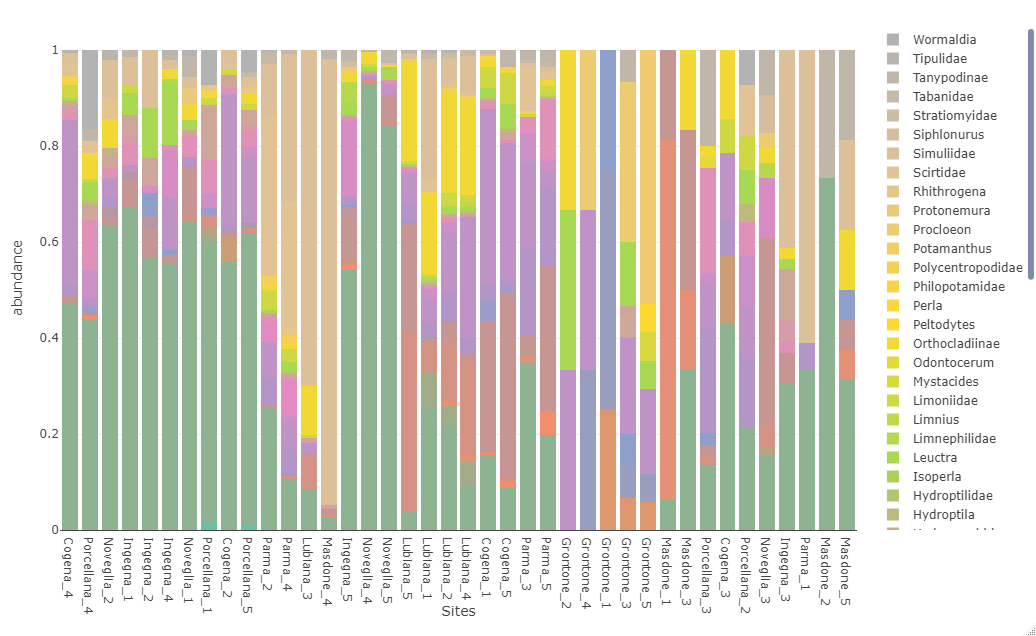


**Figure S4d** Structure of an aquatic insect community. For each observation (n = 40) the relative abundance is reported. Observations were ordered using an agglomerative hierarchical clustering with the Ward.D2 method to enhance pattern detection based on community similarity. Legend on the right is just a non-exhaustive example.

**Reference**

Sievert, C. 2020. Interactive Web-Based Data Visualization with R, plotly, and shiny. Chapman and Hall/CRC. <https://doi.org/10.1201/9780429447273>. Available: <https://plotly-r.com>

**Code used to generate the figures in SM4**

library(biomonitoR)

# load example data

data(mi_prin)

# import data into biomonitoR format

data_bio <- as_biomonitor(mi_prin, group = "mi")

# subset data to keep only insects

data_insecta_bio <- subset(data_bio, taxa = "Insecta")

# generate plot S4a

plot(data_insecta_bio)

# generate plot s4b

plot(data_insecta_bio, type ="abundance")

# generate plot s4c

plot(data_insecta_bio, type ="frequency")

# use aggregate taxa

data_insecta_agg <- aggregate_taxa(data_insecta_bio)

# generate plot S4d

plot(data_insecta_agg)
